# Secondary school principals and liminality in Polish Upper Silesia (1919-1939)

**DOI:** 10.1177/1611894421992685

**Published:** 2021-02-11

**Authors:** Machteld Venken

**Affiliations:** University of Luxembourg, Luxembourg

**Keywords:** Borderlands, history, Poland

## Abstract

Establishing and implementing rules that would teach young people to become active citizens became a crucial technique for turning those spots on the map of Europe whose sovereignty had shifted after World War I into lived social spaces. This article analyses how principals of borderland secondary schools negotiated transformation in Polish Upper Silesia with the help of Arnold Van Gennep’s notion that a shift in social statuses possessed a spatiality and temporality of its own. The article asks whether and how school principals were called on to offer elite training that would make Polish Upper Silesia more cohesive with the rest of Poland in terms of the social origins of pupils and the content of the history curriculum. In addition, it examines the extent to which borderland school principals accepted, refuted, or helped to shape that responsibility. The social origins of pupils are detected through a quantitative analysis of recruitment figures and the profiles of pupils’ parents. This analysis is combined with an exploration of how school principals provided a meaningful explanation of the recent past (World War I and the Silesian Uprisings). The article demonstrates that while school principals were historical actors with some room to make their own decisions when a liminal space was created, changed, and abolished, it was ultimately a priest operating in their shadows who possessed more possibilities to become a master of ceremonies leading elite education through its rites of passage.

The architects of Europe’s interwar set-up in Versailles in 1919 were convinced that order would be restored in Europe if the continent’s borders were redrawn.^1^ It was thought that borderland inhabitants would play a crucial role in transforming the spots on the map of Europe whose sovereignty had shifted into lived social spaces.^2^ This article investigates how establishing and implementing rules that teach children to become active citizens became a crucial technique for bringing about that transformation. Studying the history of children in a century that saw an unparalleled rise in state influence on child-rearing is increasingly recognised as a means to offer an interesting lens through which to view our knowledge of the past, since states tend to define their plans most clearly in their policies towards their future citizens.^3^ Although for a long time research tended to focus on child labour, the struggle against child mortality, and the introduction of compulsory education within single nation states, more recent enquiries have explored how children can co-create everyday life in the past.^4^

This article shifts the lens to the borderlands of interwar Polish Upper Silesia and, given the scarcity of documents produced by children themselves, investigates the practices of individual borderland secondary school principals. School principals are approached as historical actors who co-created the profile, knowledge, and capacities of the future borderland elite. I raise the question whether and how principals were called on to offer schooling that would make their region more cohesive with the rest of Poland, and examines the extent to which they accepted, refuted, or helped to shape that responsibility. This track brings the reader into the offices of borderland school principals and reconstructs how they shuffled papers on and across their tables in order to balance the different interests of the Polish government, the Silesian governor (*Voivode*), the Silesian Parliament, the Catholic Church, teachers, parents, and pupils. It is argued that school principals’ room for decision-making in determining the composition of pupils and the content of their study evolved along the sequence of Arnold Van Gennep’s rites of passage.^5^ More specifically, the anthropologist’s concept of liminality, and the way in which this concept was developed after his death, is used to identify school principals’ opportunities to influence their pupils’ profile and curriculum content.

An analytical category frequently used in recent Anglo-Saxon historiography is national indifference. It serves to unravel ‘how and why people allied themselves politically, culturally and socially from the ground up’ outside of ‘imagined national communities’.^6^ First applied to borderlands with a history in the Habsburg Empire, the category later travelled to the desks of historians dissecting Upper Silesia’s past.^7^ The historians show how nationalist efforts to transform local inhabitants into either Germans or Poles aggravated uncertainty in people about their national identifications. Despite the fact that the Catholic Church contributed to the essence of what the Polish nation stood for, just as the Protestant Church did for the German nation, local inhabitants of Upper Silesia saw religion as an alternative means of identification that enabled them to position themselves above national understandings altogether. These dynamics often remained in place when Poland regained independence; its political representatives styled it as a secular state and formulated ambitions in areas of public life that had traditionally been monopolised by the church.^8^ National indifference as an analytical category has recently been criticised because of the many contradictory convictions harboured within Upper Silesia: ‘Those who acted indifferently embraced many different “isms” and behaviours—and sometimes had little in common’.^9^ Moreover, as Tim Wilson proved, national indifference can be the cause of destructive actions and therefore should not be conceived as a desirable alternative to national identification.^10^

This article uses the analytical category of liminality in order to examine the transformation of Polish Upper Silesia through the eyes of borderland school principals. Van Gennep’s ‘rites of passage’ refer to the rituals that mark, support, or celebrate passages through the cycle of life. ‘A complete scheme of rites of passage’, Van Gennep explained, includes preliminal, liminal, and postliminal rites.^11^ The first stage harbours ‘rites of separation from a previous world’; they detach an individual or community from its former structure. The second, which Van Gennep referred to variably as ‘liminal’, ‘threshold’, or ‘transition rites’, are the rites that are ‘executed during the transitional stage’.^12^ John McKenzie later reformulated this as ‘a mode of activity whose temporal and symbolic in-betweenness allows for social norms to be suspended, challenged, played with and perhaps even transformed’.^13^ The great intensity that comes with passing through this stage of ambiguity and disorientation cannot last forever; after managing and controlling the transformation, a compliance with social norms needs to be ensured. At the moment of stabilisation, postliminal rites come into play. Through ceremonies, ‘the incorporation into the new world’ is performed.^14^

Liminality here needs to be understood in temporal and spatial terms. Most researchers consider liminality solely as having a temporality of its own, even though Van Gennep preserved a balance between time and space.^15^ In classical antiquity, the anthropologist wrote, ‘each country was surrounded by a strip of neutral ground’. Later, he would add the following:

[. . .] the same system of zones is to be found [. . .] Whoever passes from one to the other finds himself physically and magico-religiously in a special situation for a certain length of time, he wavers between two worlds. It is this situation which I have designated a transition.^16^

After Van Gennep’s death, liminality was given a broader meaning, referring to a political change and releasing forces within a society of which the dynamics and outcome are unknown. In such a case, no ‘master of ceremonies’ with experience of the entire cycle of the rite of passage is available to guide society through the change, and power, therefore, becomes highly contested.^17^

By focusing on school principals, my aim is to uncover how power and leadership played out in education during the transformation of Polish Upper Silesia. Within German-speaking schools, a teacher, and a school principal even more so, was perceived as ‘a friend, adviser and spiritual leader of the community’.^18^ Although the job was not highly paid, the fact that there was never a lack of teachers indicates that it enjoyed a certain popularity.^19^ Teachers at Polish-speaking schools, however, provoked ambiguous feelings among parents. The latter shared the teachers’ belief that more higher education graduates would be needed in order to establish a first generation of Polish-speaking intelligentsia that no longer had to assimilate to their German-speaking superiors in order to be able to advance professionally.^20^ However, in a society with neither Polish nobility nor major Polish employers, parents thought that teachers would prepare this generation to live in harmony with the working class, which turned out to be a false prediction.^21^ Already in 1932, Professor Wincenty Lutosławki noticed in Krakow: ‘I encountered my former students from Silesia later as civil servants, merchants, officers [. . .] You can see that physical work no longer charmed them’.^22^

In what follows, the impact of individual borderland school principals on the social origins of their school’s population and on the content of their curriculum is evaluated within the phases of Polish Upper Silesia’s rites of passage. Social origins are detected through a quantitative analysis of recruitment figures and the profiles of pupils’ parents. This analysis is combined with an exploration of how school principals provided a meaningful explanation of the recent past (World War I and the Silesian Uprisings). These questions are examined within the most rural and remote district of Polish Upper Silesia, Lubliniec, which covered 700 km^2^ and had 45,232 inhabitants in 1931.^23^ The article discusses six school principals from two secondary schools. The Polish-speaking school in Lubliniec was 1 of 26 secondary schools in Polish Upper Silesia to be funded with public money. Situated 30 km south of the town of Lubliniec in the neighbouring district of Tarnowskie Góry, the private German-speaking secondary school was 1 of 12 secondary schools that provided teaching in German, and 1 of 6 privately funded German-speaking schools in Polish Upper Silesia at the time.^24^ One-third of its pupils came from the Lubliniec district.^25^

## 1. The preliminal phase

The preliminal phase of Polish Upper Silesia’s rites of passage consists of the period after the end of the war until the instalment of the new political border and supranational institutions. Decision-makers in France had not reached a conclusion as to whether Upper Silesia should remain German or become Polish and pinned their hopes on a plebiscite.^26^ Between the signing of the Treaty of Versailles in June 1919 and the plebiscite in March 1921, Polish activists organised two uprisings against German rule that were quashed with the support of Entente forces. To encourage local inhabitants to vote in its favour, Germany promised to elevate the territory to the position of a separate province. In response, the Polish side issued a constitutional act that would grant the region autonomous status if it were to join Poland.

The plebiscite was the biggest experiment in self-determination in modern European history, but it did not offer a clear outcome.^27^ It was organised at a time when German and Polish national agitation encountered a local population that had yet to come to think primarily in national categories.^28^ In the Lubliniec district, 53.1% of the voters wanted to remain part of Germany, notwithstanding the fact that the last census conducted in the German Empire had indicated that 57% of the Upper Silesian population spoke Polish.^29^ Nevertheless, the League of Nations accepted the outcome.^30^ That decision fuelled a third uprising in May 1921. The region was plunged into a civil war driven by paramilitary forces fighting more out of a hunger for land and industry than on the basis of nationalist incentives.^31^ The violence that killed a thousand served to instal a line of division where previously there had been none.^32^ Finally, the League of Nations agreed that Germany would receive 71% of the Upper Silesian territory and 54% of its people, but Poland would receive the most heavily industrialised part. The Lubliniec district was cut in two: the town of Lubliniec and the areas around it were transferred to Poland, while the town of Gutentag and its surroundings remained in Germany.^33^

As Brendan Karch recently concluded, ‘the creation of ethnically based territorial nation states required more than the simple delineation of lines on a map’.^34^ The acceptance of a minority treaty with the League of Nations was made a condition for Poland’s international recognition.^35^ Under the Minority Treaty (28 June 1919), Polish citizens belonging to national minorities were entitled to use their language and to finance their schools.^36^ Polish authorities defined a minority based on what they called objective criteria, such as language. However, while the right-wing National Democrats wanted to create an ethnolinguistically homogeneous nation state in which inhabitants spoke a standardised national language without a command of other languages, a more inclusive stance towards inhabitants speaking other tongues, which was nonetheless characterised by an imperialist belief in the attractive potential of Polish culture, informed the federal agenda of Józef Piłsudski and his left-wing followers. German authorities, meanwhile, held their own subjective interpretation of minorities.^37^ The League of Nations, finally, saw itself as a protector of minority rights but lacked any powers of legal enforcement.^38^ A more elaborate treaty, the Polish–German Treaty on Upper Silesia, more commonly referred to as the Geneva Convention, was signed in 1922 for a period of 15 years. Aiming to resolve the ambiguous interpretations seeded by the Minority Treaty, it put forward a subjective definition of a minority.^39^ It prescribed, among other provisions, that a public German-speaking secondary school should be opened if supported by the guardians of 300 pupils, and made the Polish nation state provide for private secondary education in other cases.^40^ Of all the minorities living in interwar Poland, the German minority in Polish Upper Silesia was granted the most favourable conditions.

Another supranational initiative was the International Committee on Intellectual Cooperation (Comité Internationale de la Coopération Intellectuelle—hereinafter CICI), an advisory body to the League of Nations, which the Polish Ministry of Religious Denominations and Public Enlightenment (Ministerstwo Wyznań Religijnych i Oświecenia—hereinafter MWRiOP) voluntarily joined in 1924. The CICI was a forum to exchange ideas about the way in which World War I and its aftermath should be taught.^41^ Initially, it coordinated a revision of school textbooks to erase false information; later, it also issued a declaration prescribing that pupils should be given a broad historical knowledge of other nations.^42^ Polish politicians wanted to receive international money to boost intellectual life, and made sure that school books offered a pacifist narrative in return.^43^ Already by the mid-1920s, all history textbooks needed to be approved by the MWRiOP.^44^

## 2. Establishing the liminal phase

After the previous political order had come to an end, secondary school education was reoriented. The shift in national sovereignty meant that both the social origins of pupils and the content of their curriculum could no longer be taken for granted. During the establishment of the liminal phase, former traditions, hierarchies, and orders could be challenged in the Polish-speaking and German-speaking secondary schools.

We start our analysis with the two protagonists leading the Polish-speaking secondary school in Lubliniec in the first days of Polish sovereignty. Jan Szymała was born in 1889 in a poor farming family in the vicinity of Opole and received secondary schooling in Polish at a seminary near Vienna. He served as a priest in the German army during World War I and fought on the Polish side in the Third Silesian Uprising.^45^ Later, he became a priest in a Polish army unit stationed on the political border in Lubliniec, and, since the Polish state did not allow clergymen to become school principals, he worked as an administrator for the school.^46^ Polish Upper Silesia was the only region in Poland where a local decision-making body held power over education, and since the Silesian Parliament did not demand a clear separation of church and state, as was the case elsewhere in Poland, Catholic schools were able to dominate the Polish Upper Silesian interwar school landscape.^47^ This was how the priest Jan Szymała came to become a shadow school principal.^48^ Already in 1922, he transformed the German-speaking secondary school in Lubliniec into a municipal Polish-speaking school with four classes.^49^ Despite the Geneva Convention prescribing that teachers could continue their professions, Szymała dismissed the teaching staff.^50^ The municipality recruited school principals among Silesian insurgents, with no regard for whether or not they had any teaching experience, and gave them short-term contracts. One of them, Wiktor Bazanowski, was the school principal when the first archived document was produced in 1924.^51^

Jan Szymała and Wiktor Bazanowski were determined to bring socially underprivileged children to the school and raise enough funds to pay for their education.^52^ The Silesian Parliament and the Lubliniec town council offered modest financial support, but Szymała and Bazanowski decided to go further.^53^ The fact that the school’s cash book contained more red than black figures shows that a majority of parents could not pay the tuition fee, but that their children were nevertheless able to continue their education.^54^ The duo also put forward their own interpretation of the past. Given the absence of clear guidelines from the Silesian Department of Education, and despite the MWRiOP requiring history textbooks to promote peace, in a town where a majority of people had voted to remain in Germany, the two insurgents spread the message that the Silesian Uprisings, not the war or the plebiscite, legitimised the borderland’s shift to Polish sovereignty.^55^ This myth was developed and promoted by the biggest political party in Silesia, the Christian Democrats, to which both insurgents most probably belonged. Johanna Haubold-Stolle explained,

the insurgents were stylised as local heroes, who not only fought with heroism against the foreign, barbarian oppressors of Upper Silesia, but also fought for the freedom of at least a part of Upper Silesia [. . .] In that mythical representation, the uprisings were the real plebiscite.^56^

As the teaching staff in Polish Upper Silesia were deeply divided between those who had migrated to Lubliniec and locally recruited teachers, Bazanowski ordered his secretary to write the following in the protocol of his first staff meeting: ‘The school principal wishes to state that he has taken care of the books and regulations of the directorate. He asks for them to be signed and applied strictly. Comments and polemics are not allowed in the announcement book’.^57^ Bazanowski pushed away the pacifist narrative, as well as any possible discussions about an alternative. Since Polish history textbooks were largely unavailable at the time, his measure was probably quite effective.^58^

The liminal phase established in the German-speaking private secondary school in Tarnowskie Góry took a different shape. The Geneva Convention legally defined and supranationally controlled a liminal phase in Polish Upper Silesia’s education.^59^ The three surviving annual reports written by school principal Josef Czaja—an individual about whom we know nothing other than that he had a Silesian family name—during his service between 1922 and 1932 show that there were no significant changes in the social origins of pupils during this period. In 1931, the 184 pupils at the school had parents working as skilled specialists in local industry (more than 50 parents) or as craftsmen (26), but not as day labourers. Only four of the pupils received a stipend from the Deutsche Schulverein in Katowice, the institution coordinating German minority schooling in Polish Upper Silesia, which was funded by German taxpayers’ money.^60^ The fact that few pupils finished their education—in 1932, eight pupils from the school participated in the school-leaving exam organised by the MWRiOP, and just six passed—indicates that the education was something of a dead end.^61^

The same was true for what pupils needed to learn about their past. Although already in 1922, as Anna Novikov observed, the Department of Education chose history ‘as the most important subject in order to start promoting among the German-speaking children the new ideology of the Polish State’, Josef Czaja managed to postpone the implementation of this strategy.^62^ He could get away with merely indicating the hours of history teaching and mentioning that the school possessed historical wall maps in his yearly reports.^63^ Moreover, the spokesperson of the Deutsche Schulverein, Paul Poralla, would later confess that ‘skilful manoeuvring’ in meeting the demands for teaching enabled the staff of German-speaking schools in the period up to 1932 not to ‘damage the special mission to educate German people’.^64^

## 3. Within the liminal phase

During the establishment of the liminal phase, principals of both schools had considerable opportunities to determine the profile of pupils and the content of the curriculum; however, once in the liminal phase, their decision-making power diverged. Within the liminal phase, Van Gennep noted ‘certain instances where the transition possesses an autonomy of its own as a secondary system within a ceremonial whole’.^65^ Although such secondary systems existed in both schools, their arrangement differed. The heads of the Polish-speaking school determined the content of history teaching for a largely self-selected group of pupils. In the German-speaking school, however, Polish authorities successfully managed to limit that secondary system to the school’s clientele.

In 1926, the town council appointed Józef Arecki as the new principal of the Polish-speaking secondary school. He had worked as a teacher in the former region of Galicia, held a PhD in Philosophy from the University of Lemberg, and had come to Upper Silesia to supervise the propaganda activities of a Polish-minded group in the run-up to the plebiscite.^66^ Arecki extended the four-year school curriculum by an extra year, but the town council was convinced that a fifth year would only benefit a small number of pupils and proceeded to fire Arecki.^67^ The new governor Michał Grażyński annulled that decision, however, because he wanted to harmonise school curricula.^68^ Grażyński was supportive of the Sanacja regime, which had come to power after Piłsudski staged a political coup promising to save politics from corruption. The school was taken out of municipal hands and became a state school named after the Romantic poet Adam Mickiewicz. This decision had far-reaching consequences. Municipal scholarships dried up, children of immigrant Polish civil servants were granted privileged access, and the school closed its doors to girls.^69^ When the parents of 60 girls pleaded for their daughters to be allowed to attend the state school, they were told that the compulsory primary education of 1300 mostly poor children needed to be prioritised over the secondary education of a small number of girls from wealthy families.^70^

Jan Szymała opposed the change and found a way to diversify the school’s clientele. His solution enabled him to continue his revolutionary mission with the approval of the Voivodeship.^71^ Szymała recruited pupils from rural families from the area of his birth, which had ended up on the German side. He brought them to Lubliniec for secondary school teaching in Polish that was lacking in German Upper Silesia.^72^ By 1930, the number of pupils from Germany had increased to about half of the school population, and their fees were covered by the Voivodeship.^73^ The school’s yearly report showed that 210 boys attended, and their parents’ professions were specified as follows: 79 farmers, 38 blue-collar workers, and just 41 civil servants.^74^ The considerable variety in professional backgrounds was a result of Szymała’s initiative.^75^

Because of the presence of migrant pupils, it was decided that the school curriculum should parallel the secondary school curriculum in Germany.^76^ Grażyński forced other state schools to bring the number of teaching hours and the content of history lessons into line with the Polish ministerial teaching plan. The MWRiOP also continued to carefully check narratives of the recent past in history textbooks to ensure that they included tolerance towards national minorities.^77^ Notwithstanding this, not a word about history can be found in the sole remaining yearly report from that period, written in 1930 by Arecki and containing more than 60 pages. In the school library, moreover, history books accounted for less than 5% of the total collection.^78^ History was not considered a useful subject for coming to terms with the past. At the Adam Mickiewicz school, children were to be taught about the past through Polish classical literature rather than history or Silesian myths.

Within the German-speaking school, Van Gennep’s secondary system only consisted of the school’s autonomy in selecting pupils, and the social origins of the pupils did not change. As long as parents were willing to pay, Polish nationalists could not challenge the bourgeois profile of German-speaking elite pupils. At the same time, the school gradually lost its say over the content of its teaching. When Hans Klemenz, who was born in Silesia in 1894 to a secondary school teacher and who trained at the Universities of Breslau and Munich, became the school principal in 1934, the National Socialists’ seizure of power in Germany had already subverted the international mechanism of minority protection, and Germany had started to aspire explicitly to the re-annexation of Polish Upper Silesia.^79^ The Sanacja leadership in Warsaw reacted to these developments by annulling the Minority Treaty and signing a bilateral German–Polish non-aggression pact in 1934.^80^ Nevertheless, the Geneva Convention remained in place in order to safeguard the legally defined liminal phase for German-speaking education in Polish Upper Silesia.

Polish nationalists started to attack history teaching at the German-speaking school. The MWRiOP forbade the staff from teaching about the World War and its aftermath, thereby silencing the master narrative among German-minded Silesians presenting Upper Silesia as a bleeding wound caused by the redrawing of the political border, an image which had even come to symbolise the suffering of Germany as a whole.^81^ It also considered German history textbooks covering anything from the fourteenth century onwards inappropriate, because their narrative of lands inhabited by German-speaking people clashed with the perceived Polish character of Silesia and Piłsudski’s leading role in Poland’s struggle for independence.^82^ In 1933-1934, a Polish school inspector pointed to the insufficiently Polish character of history teaching. A year later, Hans Klemenz listed in the annual report the history teaching materials that were officially required in German-speaking schools (history textbooks in Polish approved by the MWRiOP and a collection of history sources compiled by the Deutsche Schulverein), and stated that pupils had attended public parades organised on Polish state holidays, including Armistice Day (11th November).^83^ Between the lines, he admitted to not always having been able to keep his pupils under control: ‘The pupils have been constantly urged to be a credit to the school by presenting themselves well in the public sphere’.^84^

His pupils felt attracted by the kind of Reich membership that the National Socialists in Germany granted them out of a belief that people’s blood was more important than their citizenship.^85^ With the help of the gymnastics teacher Edward Bittmann, the pupils had set up a hiking club (*Wanderbund*) modelled on the Nazi youth organisation Hitlerjugend and containing several branches throughout Polish Upper Silesia.^86^ After attending the inauguration of a commemorative stone for German soldiers fallen during the war, they were accused of having declared their loyalty to Adolf Hitler; 49 members were arrested, 27 adults were sentenced to prison, and 15 youngsters between the ages of 15 and 17 were sent to a reformatory.^87^ School principal Klemenz was accused of having allowed his pupils to meet alumni outside the school and engage in organised activities with them, despite a ministerial regulation of 1927 stipulating that youth organisations could operate only under the supervision of a school principal or a religious order.^88^ Klemenz testified, ‘In a small town, it is very difficult to ensure without any doubt whether unavoidable contact and the chance of meetings between pupils and former pupils goes beyond the scope of personal contact and takes on an organised form’.^89^ He pleaded in vain and was dismissed.

## 4. (Towards) the postliminal phase

Later in the 1930s, the liminal phase in Polish Upper Silesia came to an end. A new stability was attained in which borderland secondary schooling was entirely incorporated into the Polish educational system. Sources that we possess shed light on the role of school principals during the ceremony inaugurating the postliminal phase in the Polish-speaking school, as well as during the closure of the liminal phase in the German-speaking school.

Although Polish Upper Silesia enjoyed autonomy in educational measures and Sanacja supporters never achieved a majority in the Silesian Parliament, in 1932, Grażyński took advantage of a careless formulation in the founding document to strip the Voivodeship of its autonomy in most fields related to education and to bypass the decision-making capacity of the Silesian Parliament.^90^ As a result, the curriculum of the Polish-speaking school was brought into line with Polish secondary education, consisting of four years of middle school and two years of high school.^91^ That the school’s migrant pupils could continue their education in the newly founded Polish-speaking secondary school in German Upper Silesia was an additional reason for the homogenisation of the curriculum.^92^

The role of religion, however, continued to differ from elsewhere in Poland. Other Polish schools needed to take a stance somewhere between the Polish Minister of Education Janusz Jędrzejewicz’s opinion that religion should not influence the functioning of public institutions, and the ongoing campaign of the Catholic Church against non-religious education, strengthened by a statement by Pope Pius XI in 1929 against educational neutrality.^93^ The Polish-speaking school continued to function as a publicly funded Catholic school until 1939, when all Catholic schools in Polish Upper Silesia were renamed public schools.^94^

**Figure 1.**
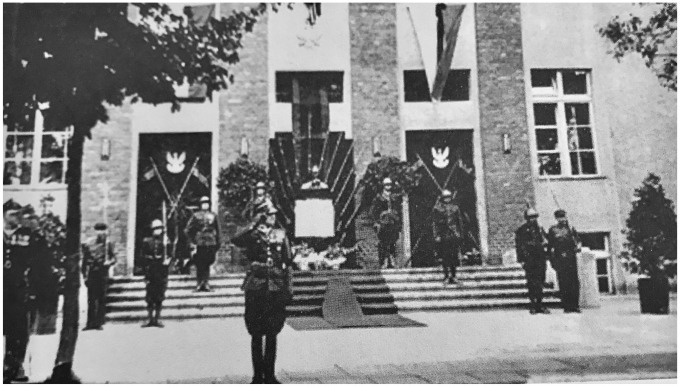
When Marshal Józef Piłsudski died in 1935, a symbolic funeral ceremony was held in front of the Polish-speaking secondary school in Lubliniec.^98^

After the death of Józef Piłsudski in 1935, Poland began to be run autocratically by people who had closely cooperated with the former marshal and endorsed the myth surrounding the ex-combatants whose military efforts had brought about the resurrection of the state in 1918.^95^ Civic education was gaining the upper hand over history teaching, and pupils were taught to obey the army and the police rather than the government.^96^ In the Polish-speaking school, pupils were taught how they could participate in ruling their undemocratic country. Scarce documentation makes it difficult to trace how the new school principal Adam Tyran (1932–1939)—born in the vicinity of Kraków and a teacher in Upper Silesia from 1923—and Jan Szymała influenced the composition of pupils, but there is empirical evidence that 120 pupils received a final certificate between 1930 and 1939, and that the school opened its door to girls again in 1932.^97^ There is also a picture displaying how historical knowledge was narrated (Figure 1). It depicts the symbolic funeral ceremony following a military parade held in front of the school building to mark the death of Marshal Józef Piłsudski.

Throughout Poland, the idea spread that the independent Polish state had not been a chance outcome of international negotiations but had been achieved by the toil and sweat of the successful leader of the Polish Legions.^99^ Pupils in Poland were increasingly engaged in performing history during ceremonies and parades rather than learning it with the help of history textbooks. Paradoxically, the Polish government was very active in promoting a pacifist interpretation of the Great War in school textbooks. At the International Conference on Disarmament in 1932, the Polish delegation presented a draft convention prohibiting activities intended to disrupt good relations between peoples.^100^ As a result of the conference, any National Committee on Intellectual Cooperation could call for a different interpretation of history by indicating to another country’s National Committee if it felt that school textbooks printed within that country presented false information or offered biased commentaries.^101^ This included the German National Committee, since the CICI continued its cooperation with Germany after it left the League of Nations, believing that intellectual cooperation with a totalitarian regime was possible. The Polish National Committee feverishly investigated the narrative of the war in more than 300 school textbooks published abroad, and labelled 63 of them ‘extraordinarily dangerous for international solidarity’.^102^ At the same time, it did not formulate objections against a single textbook published in Poland.^103^ In 1937, Poland signed a declaration for the revision of school textbooks distributed by the Secretary General of the League of Nations a year earlier, thereby agreeing to facilitate a broad historical knowledge of other nations and protect against a tendentious presentation of historical events.^104^ Moreover, on the initiative of Poland, a bilateral German–Polish commission on the revision of textbooks met in Berlin and Warsaw in 1937.^105^ There were enough people in Poland who knew how to offer a pacifist narrative of the World War, but these people were overruled.

School principal Adam Tyran and school administrator Jan Szymała took several steps to incorporate the regional context of Polish Upper Silesia into the preset national norms on history teaching.^106^ They invited the 74th Polish Upper Silesian Infantry Regiment, a military unit monitoring the political border, to participate in the Piłsudski ceremony. This ceremony demonstrated their regional interpretation of the national narrative on war memory at the time, even though history textbooks said something different. The ceremony presented elite pupils as successors of two traditions that would henceforth be intertwined: the Polish Legions and the Polish heroes battling in the Silesian Uprisings. The regiment indicated to pupils that Piłsudski’s tradition needed to be continued; the border was to be defended.^107^ More than half a century later, former pupil Wladyslaw Liszkowski still remembered the event: ‘A military parade was held for the entire 74th Infantry Regiment. This was a beautiful celebration that had a great impact on our national and patriotic sense’.^108^

The sources that we possess about the German-speaking school offer us an insight into how the liminal phase came to an end. In 1937, the Geneva Convention was replaced by a Polish–German agreement pledging mutual respect. Polish authorities could now influence the profile of pupils, as the choice for a suitable school for German-speaking children no longer lay with the children’s guardians but with ‘the will of Polish society, which will condemn once and for all those who are traitors of the national cause’.^109^ In 1936, the Polish Minister for Education, Wojciech Alojzy Świętosławski, anticipated the termination of the Convention by requiring German-speaking secondary schools to teach the full history curriculum of Polish-speaking schools. Believing that German history should fulfil ‘the axis of education’, the Deutsche Schulverein responded by proposing a plan to the Voivodeship for history teaching based on self-composed teaching materials. These granted a central place to World War I and the Silesian Uprisings, and compared dominant German and Polish narratives.^110^ The Voivodeship administration, however, ruled that pupils in German-speaking classes should not devote more time to these topics than pupils in Polish-speaking classes, and that the content needed to be reduced, but did not provide clear guidelines. In 1937, Polish school inspectors wrote their first evaluations based on the Voivodeship’s requirements. Their observations led to the Voivodeship administration deciding that two of the six private German-speaking secondary schools in Polish Upper Silesia, including the school in Tarnowskie Góry, provided an ‘insufficient level of education’ and needed to be closed.^111^

The Director of the Deutsche Schulverein intended to lodge an appeal with a Polish court to oppose the Voivodeship’s decision. Because the appeal needed to be filed by school principals, Paul Thomalla, the new principal in Tarnowskie Góry, was invited to defend the school’s history teaching. He did everything that he could to avoid the task. He twice showed up for a meeting with the Deutsche Schulverein without a draft, and later confirmed over the phone that he would not send anything. The private German-speaking school in Tarnowskie Góry was shut down soon afterwards.^112^

Shortly after the German annexation of the Sudetenland in October 1938, Hitler demanded the annexation of the Free City of Danzig and a connecting road to East Prussia through the Polish Corridor, but he had not yet intended to interfere with the sovereignty of Polish Upper Silesia. Nevertheless, at the beginning of the 1938-1939 school year, teachers of German-speaking schools in Polish Upper Silesia were required to sign an oath of loyalty to the Polish state for the first time.^113^ Poland was attacked by the German army in September 1939.

## 5. Conclusion

Arnold Van Gennep’s rites of passage enable us to identify how much room for decision-making was afforded to principals of borderland schools during Polish Upper Silesia’s transformation in the interwar period to (co-)determine which children were to become members of the borderland elite and what borderland secondary school pupils were expected to know about their past. School principals were historical actors who gave meaning to the space and time in which the transformation of social statuses was negotiated, accomplished, and regulated.

The founding principles of the Polish nation state were a decisive factor in determining how much room for decision-making principals of the Polish-speaking secondary school in Lubliniec possessed. They were granted a great deal of freedom to change the bourgeois profile of elite teaching, because Polish state officials and Silesian administrators firmly believed that if Polish-speaking children were to identify with the Polish state, they would be offered paths to social advancement. In setting the agenda of historical knowledge about the recent past, on the contrary, options ranging from a mythologised interpretation of the Silesian Uprisings to teaching history through classical literature could only exist as long as Polish state officials did not extend their realm of control to the school. Once the Sanacja regime had erased the liminal phase, the only possible variation appeared to be a regionalist addition to the regime’s narrative of war memory. In the German-speaking school in Tarnowskie Góry, liminality was defined by the supranational control mechanism of the League of Nations. It created a space and time in which the bourgeois social composition of the school could not be challenged by Polish nationalists, but neither could it impede these nationalists in their attempt to increase their say over the school’s curriculum. School principal Hans Klemenz, for example, was dismissed for challenging the Polish master narrative on war memory. Furthermore, once the liminal phase had come to an end, the German-speaking school was forced to close its doors.

In conclusion, the pupils of Polish Upper Silesia’s interwar secondary schools were children of transformation. Most of them received their training during a period of time when elite education no longer belonged to the old world but had not yet been fully incorporated within the new world. In the in-between world, power was highly contested and school curricula changed as frequently as the composition of the schools’ clientele. Within the system of power that was in place at the time, only a figure in the shadows managed to turn himself into a liminal subject, finding creative solutions and negotiating changes that helped to shape elite education. School administrator Jan Szymała from the Polish-speaking school was capable of negotiating his understanding of elite training by jumping in and out of a liminal phrase depending on the changing political context. Understanding him as a ‘master of ceremonies’ of Polish Upper Silesia’s rites of passage, rather than identifying him as an indifferent Catholic national school administrator, offers the advantage of highlighting the importance of space and time. It enables us to dissect his practices within a sequence of transformative events that inevitably led to compliance with social norms. These practices were articulated within a space considered to be of such crucial interest to Polish nationalists that placing oneself outside of the imagined national community, or dictating its meaning, was no longer an option.

## Notes

1. M. MacMillan, *Paris 1919. Six Months that Changed the World*, New York 2002, 221.

2. C. Mezger, *Forging Germans. Youth, Nation, and the National Socialist Mobilization of Ethnic Germans in Yugoslavia, 1918–1944*, Oxford 2020; M. Venken (ed.), *Borderland Studies Meets Child Studies. A European Encounter*, Frankfurt am Main 2017; T. Zahra, *Kidnapped Souls. National Indifference and the Battle for Children in the Bohemian Lands 1900–1948*, Ithaca, London 2008.

3. M. Saxton, ‘Introduction into the First Volume of the Journal of Childhood and Youth’, in: H. Morrison (ed.), *The Global History of Childhood Reader*, New York 2012, 104; D. Schumann, *Raising Citizens in the ‘Century of the Child’. The United States and German Central Europe in Comparative Perspectiv*e, New York 2010.

4. P. N. Stearns, ‘Challenges in the History of Childhood’, in: *The Journal of the History of Childhood* 1 (2008), 35–42.

5. A. Van Gennep, *The Rites of Passage*, Chicago 1960.

6. T. Zahra, ‘Imagined Non-Communities. National Indifference as a Category of Analysis’, in: *Slavic Review* 69 (2010) 1, 93–119, 118.

7. P. Judson, *Guardians of the Nation. Activists on the Language Frontiers of Imperial Austria*, Cambridge 2006.

8. J. Bjork, *Neither German nor Pole. Catholicism and National Indifference in a Central European Borderland*, Ann Arbor 2008; B. Porter, ‘Thy Kingdom Come. Patriotism, Prophecy and the Catholic Hierarchy in Nineteenth-Century Poland’, in: *Catholic Historical Review* 89 (2003) 2, 213–239, 238.

9. B. Karch, *Nation and Loyalty in a German-Polish Borderland. Upper Silesia, 1848–1960*, Cambridge 2018, 17.

10. See P. Polak-Springer’s review of T. Wilson’s book: T. Wilson, *Frontiers of Violence. Conflict and Identity in Ulster and Upper Silesia 1918–1922*, New York 2010; P. Polak-Springer, ‘Violence in Europe’s Peripheries in the Aftermath of World War I’, in: *H-Poland* 1–4 (2015), 20 September 2017.

11. Van Gennep, *Rites*, 11.

12. Idem, 21, 186.

13. J. McKenzie, ‘The Liminal-Norm’, in: H. Bial (ed.), *The Performance Studies Reader*, London, New York 2004, 26–31, 27.

14. Van Gennep, *Rites*, 21.

15. E. Karaboeva, ‘Borders and Go-betweens. Bulgarian International Truck Drivers during the Cold War’, in: *East Central Europe* 41 (2014) 2–3, 223–253, 230.

16. Van Gennep, *Rites*, 17-18.

17. B. Thomassen, ‘The Uses and Meanings of Liminality’, in: *International Political Anthropology* 2 (2009) 1, 5–28, 22, 51.

18. Anonymous, ‘Der Lehrer und sein Dorf’, in: *Deutsche Schulzeitung* 18 (1937/1938), 16.

19. I. Eser, *‘Volk, Staat, Gott!’. Die deutsche Minderheit in Polen und ihr Schulwesen 1918–1939*, Wiesbaden 2010, 508.

20. M. W. Wanatowicz, *Inteligencja na Śląsku w okresie międzywojennym*, Prace Naukowe Uniwersytetu Śląskiego w Katowicach, vol. 762, Katowice 1988, 8, 16.

21. E. Kopeć, *‘My i oni’ na polskim Śląsku (1918–1939)*, Katowice 1986, 25, 42–43.

22. W. Lutosławski, ‘Moja praca na Śląsku’, in: *Zaranie Śląskie* (1932), 132.

23. M. Venken, *Peripheries at the Centre. Borderland Schooling in Interwar Europe*, New York 2021; Główny Urząd Statystyczny Rzeczypospolitej Polskiej, *Mały rocznik Statystyczny*, Warsaw 1937, 15.

24. J. Brey, ‘Das deutsche höhere Schulwesen in Polnisch-Oberschlesien’, in: V. Kauder (ed.), *Das Deutschtum in Polnisch-Schlesien. Ein Handbuch über Land und Leute*, Leipzig 1932, 303–306.

25. Archiwum Państwowe w Katowicach, 78/19 (Jahresbericht des Privaten human. Gymnasiums mit deutscher Unterrichtssprache in Tarnowskie-Góry für das Schuljahr 1931/1932), 31–32. On the Seminary of the Religious Order of the Oblates in Lubliniec, one of two Polish-speaking private schools operating in Polish Upper Silesia, see: J. Olszówka, ‘Szkolnictwo polskie w powiecie lublinieckim w latach 1922–1939’, in: J. Pluta (ed.), *Nauczyciele Ziemi Lublinieckiej w służbie Polskiej Oświacie, Biuletyn Metodyczny. Związek Nauczycielstwa Polskiego*, Katowice 1979, 11–29, 25.

26. C. Fink, *Defending the Rights of Others. The Great Powers, the Jews, and International Minority Protection, 1878–1938*, Cambridge, New York 2004, 135–140, 235.

27. J. Bjork / T. Kamusella / Novikov T. / Wilson A., ‘Introduction’, in: T. Kamusella / J. Bjork / T. Wilson */* A. Novikov (eds.), *Creating Nationality in Central Europe, 1880–1950. Modernity, Violence and (Be)Longing in Upper Silesia*, London 2016, 1–7, 1.

28. W. Lesiuk, ‘Die Entstehung und Entwicklung des deutsch-polnischen Konflikts in Oberschlesien zu Beginn des 20. Jahrhunderts’, in K. Struve (ed.), *Oberschlesien nach dem Ersten Weltkrieg. Studien zu einem nationalen Konflikt und seiner Erinnerung*, Marburg 2003, 1–32.

29. S. Wambaugh, *Plebiscites since the World War. With a Collection of Official Documents*, Washington 1933, I 211; A. Rodriguez, ‘“Scoundrels” and Desperate Mothers: Gendering German and Polish Propaganda in the Upper Silesian Plebiscite, 1919–1921’, in: T. Kamusella / J. Bjork / T. Wilson */* A. Novikov (eds.), *Creating Nationality*, 87–105, 100.

30. A. Prażmowska, *Poland. A Modern History*, London 2013, 93–94.

31. Bjork, *Neither German*, 256.

32. Wilson, *Frontiers*, 213.

33. J. Jaros (ed.), *Lubliniec. Zarys rozwoju powiatu*, Katowice 1972, 9.

34. Karch, *Nation*, 302.

35. S. Zloch, *Polnischer Nationalismus. Politik und Gesellschaft zwischen den beiden Weltkriegen*, Cologne 2010, 168.

36. C. Raitz von Frentz, *A Lesson Forgotten. Minority Protection under the League of Nations. The Case of the German Minority in Poland. 1920–1934*, Hamburg 1999, 265.

37. T. Kamusella, ‘The Isomorphism of Language, Nation, and State: The Case of Central Europe’, in: W. Burszta / T. Kamusella / S. Wojciechowski. (eds.), *Nationalisms across the Globe: An Overview of Nationalisms of State-Endowed and Stateless Nations, vol. 2: The World*, Poznań 2006, 57–92, 57, 79; A. Novikov, *Shades of a Nation. The Dynamics of Belonging among the Silesian and Jewish Populations in Eastern Upper Silesia (1922–1934)*, Osnabrück 2015, 204.

38. MacMillan, *Paris*, 97.

39. K. Junckerstorff, *Das Schulrecht der deutschen Minderheit in Polnisch-Oberschlesien nach dem Genfer Abkommen*, Berlin 1930.

40. Raitz von Frentz, *A Lesson Forgotten*, 266.

41. A. M. Brzeziński, ‘Karol Lutostański – prezes Polskiej Komisji Międzynarodowej Współpracy Intelektualnej (1924–1939)’, in: *Przegląd Nauk Historycznych* 1 (2004) 5, 107–119, 107.

42. A. M. Brzeziński, *Polska Komisja Międzynarodowej Współpracy Intelektualnej (1924–1939)*, Łódź 2001, 87.

43. A. M. Brzeziński, ‘Polska w systemie międzynarodowej współpracy intelektualnej Ligi Narodów (1922–1939)’, in: *Dzieje Najnowsze* 34 (2002) 2, 3–22, 3.

44. H. Pohoska, ‘Poland’, in: Working Committee of a Special Commission on Education Stockholm (ed.), *Report on Nationalism in History Textbooks*, vol. 1, Stockholm 1928, 201–217.

45. S. Kowalewski, ‘Ksiądz pułkownik Jan Szymała, czyli o potrzebie pamięci’, in: *Lubliniecki pamiętnik pedagogiczny*, 2 (1997), 8–17.

46. APK, 27/200, 8 (Ustawa w sprawie rozciągnięcia na obszar Województwa Śląskiego mocy obowiązującej niektórych przepisów ustawy z dnia 11 marca 1932 r. o ustroju szkolnictwa).

47. M. W. Wanatowicz, ‘Województwo śląskie (1922–1939)’, in: J. Bahlcke / D. Gawrecki / R. Kaczmarek (eds.), *Historia Górnego Śląska. Polityka, gospodarka i kultura europejskiego regionu*, Gliwice 2011, 238–248; APK, 1376/1086 (Uchwała Rady Miejskiej w Lublińcu z dnia 3.3.1933 r.), 70.

48. L. Markowiak, ‘Stanisław Książek (1908-1980)’, in: *Lubliniecki Pamiętnik* 3 (2003), 132–138, 133.

49. Loben-Guttentager Kreisblatt 16 (09/12/1971), 2.

50. Gemischte Kommission für Oberschlesien, *Amtliche Sammlung der Stellungnahmen des Präsidenten der Gemischten Kommission für Oberschlesien auf dem Gebiete des Minderheitenrechtes auf Grund der Vorschriften des III. Teils des deutsch-polnischen Genfer Abkommens vom 15. Mai 1922 in der Zeit vom 15. Juni 1922 bis 15. Juli 1937*, vol. 1, Berlin 1937, 68–86.

51. APK 1376/1211 (Rota ślubowania, Lubliniec, 18.1.1926), 258.

52. M. Janik, ‘Zespół Szkół nr 1 im. Adama Mickiewicza w Lublińcu. Dzieje Szkoły w latach 1922–2012’, in: *Lubliniecki Pamiętnik Pedagogiczny* 4 (2012), 5–71, 10.

53. S. Górski, *Organizacja i rozwój szkolnictwa oraz ich uwarunkowania społeczno-polityczne w świetle działalności Sejmu Śląskiego (1922-1939)*, Katowice 1978, 197.

54. APK, 1376/1130 (Świadectwo niezamożności, 24.10.1924), 570; APK, 1376/1130 (Opłata szkolna i wstępna na czas od 1 września do 31 grudnia 1922), 507–512, 511.

55. A. Novikov, *Shades of a Nation. The Dynamics of Belonging among the Silesian and Jewish Populations in Eastern Upper Silesia (1922–1934)*, Osnabrück 2015, 46; A. Michalczyk, *Heimat, Kirche und Nation. Deutsche und polnische Nationalisierungsprozesse im geteilten Oberschlesien 1922–1939*, Köln, Weimar 2010, 74, 81.

56. J. Haubold-Stolle, *Mythos Oberschlesien: Der Kampf um die Erinnerung in Deutschland und in Polen 1919–1956*, Osnabrück 2008, 242.

57. APK, 1376/1211 (Protokół z pierwszego posiedzenia rady pedagogicznej, Lubliniec, 28.10.1924), 57.

58. Idem, 59.

59. Junckerstorff, *Schulrecht*, 54–58.

60. APK, 78/19 (Jahresbericht 1931/1932), 31-32; See also M. Kneip, *Die deutsche Sprache in Oberschlesien. Untersuchungen zur politischen Rolle der deutschen Sprache als Minderheitensprache in den Jahren 1921–1998*, Dortmund 2000, 83.

61. APK, 78/19 (Jahresbericht 1931/1932), 14.

62. Novikov, *Shades*, 66.

63. APK, 78/6 (Jahresbericht des Privaten human. Gymnasiums mit deutscher Unterrichtssprache in Tarnowskie-Góry für das Schuljahr 1922/1923); Idem, 78/7, (Jahresbericht des Privaten human. Gymnasiums mit deutscher Unterrichtssprache in Tarnowskie-Góry für das Schuljahr 1924/25); idem, 78/19 (Jahresbericht 1931/32), 13–14.

64. Paul Poralla quoted in Eser, *Volk*, 569.

65. Van Gennep, *Rites*, 185.

66. APK, 1376/1211, (Odpis, Curriculum vitae, Dr. Józef Jan Arecki, Przemyśl, 7.7.1924), 271.

67. Idem (Wydział Wykonawczy Rady Rodzicielskiej Gimnazjum Miejskiego w Lublińcu do wydziału Oświecenia Publicznego w Katowicach, Lubliniec, 30-5.1925), 484; idem (Magistrat i Rada Miejska miasta Lublińca do wojewody śląskiego, Lubliniec, 22.6.1926), 487.

68. Idem (Województwo Śląskie, Wydział Oświecenia Publicznego do Magistratu w Lublińcu, Katowice, 30.6.1926), 506–507.

69. Idem, 1376/1130 (Magistrat Lubliniec, Lubliniec, 21.5.1926), 671.

70. Idem, 1376/1211 (Do Magistratu w Lublińcu, Lubliniec, 6.8.1929); ibid., 46 (Protokół zebrania rodziców w dniu 14 maja 1929, Lubliniec, 14.5.1929), 44; idem (Do Magistratu w Lublińcu, Lubliniec, 13.8.1930), 64.

71. Kowalewski, ‘Ksiądz’, 12.

72. *‘17.1.1927: Leitendes Grenzkommissariat Oppeln an den Oberpräsidenten ebendort. Polnische Bewegung im Kreis Oppeln. (AP Opole. Naczelne Prezydium Prowincji Gornoslaskiej w Opolu, nr. 79)’*, in: R. Jaworski / M. Wojciechowski (eds.), *Deutsche und Polen zwischen den Kriegen. Minderheitenstatus und ‘Volkstumskampf’ im Grenzgebiet. Amtliche Berichterstattung aus beiden Ländern 1920–1939.* 2. Halbband. München 1997, 832–834.

73. Janik, ‘Zespół’, 5.

74. ‘Roczne Sprawozdanie Dyrekcji’, in: *Biblioteka Śląska w Katowicach, zbiory specjalne* (1931), 56–57.

75. ‘Praca wychowawcza szkoły w roku szkolnym 1930/31’, in: *Lubliniecki Pamiętnik Pedagogiczny* 3 (2003), 33–38.

76. L. Markowiak, ‘Lublinieckie Gimnazjum w latach II Rzeczypospolitej’, in: *Lubliniecki pamiętnik pedagogiczny* 1 (1994), 59.

77. Brzeziński, *Komisja*, 77.

78. ‘Roczne Sprawozdanie Dyrekcji’, 23.

79. Dokumentesammlung des Herder-Instituts Marburg, Sammlung Jendrike (Formular Hans Klemenz, 1956); N. J. Cull, ‘Anti-Semitism’, in: N. J. Cull / D. H. Culbert / D. Welch (eds.), *Propaganda and Mass Persuasion. A Historical Encyclopedia. 1500 to the Present*, Santa Barbara 2003, 13–15, 14; M. Allinson, *Germany and Austria 1814–2000. Modern History for Modern Languages*, New York 2002, 89.

80. I. L. Claude, *National Minorities. An International Problem*, Harvard 1955, 30.

81. Haubold-Stelle, *Mythos*, 160; Bundesarchiv Berlin-Lichterfelde, DS, 644 (Poralla, P., Die deutschen Unterrichtsprogramme, die Stellung der Wojewodschaft dazu und die daraus sich ergebenden Massnahmen, 2.2.1936), 53.

82. R. Gelles, *Sprawy polskie w szkole niemieckiej w latach 1919–1939*, Wrocław 1991, 101, 108–109, 113.

83. APK, 78/20 (Jahresbericht des Privaten human. Gymnasiums mit deutscher Unterrichtssprache in Tarnowskie-Góry für das Schuljahr 1934/1935), 15.

84. APK, 78/20 (Jahresbericht 1934/35), 29.

85. W. Chu, *The German Minority in Interwar Poland*, Cambridge 2012, 278.

86. Eser, *Volk*, 583.

87. Politisches Archiv des Auswärtigen Amts Berlin, P 17 (Politischer Bericht, Kattowitz, 7.12.1936); Idem, R 104054 (Deutsches Generalkonsulat, Flüchtlinge der NSDAP in Deutsch-Oberschlesien, Kattowitz, 25.6.1936); T. Bierschenk, ‘III. 1. Im Schatten des Nichtangriffspaktes (26.1.1934 -14.7.1937), Die Deutsche Volksgruppe in Polen 1934-1939’, undated, www.wintersonnenwende.com (accessed 25 March 2020); Kneip, *Die deutsche Sprache*, 105.

88. P. Greiner, *Polski ruch młodzieżowy w województwie śląskim w latach 1922–1939*, Wrocław 1993, 33.

89. BABL, R 8043/644 (Einspruch des bisherigen Direktors Hans Klemenz vom Deutschen Privatgymnasium Tarnowskie Góry gegen seine Enthebung vom Leiterposten 17.1.1936), 281.

90. A. Glimos-Nadgórska, *Polskie szkolnictwo powszechne województwa śląskiego (1922–1939)*, Katowice 2000, 112; Górski, *Organizacja*, 75; APK 27, 200F (*Śląsk złączony z Macierzą musi mieć jednolity z Polską ustrój szkolny*), ‘Polska Zachodnia’, 12 March 1933), 354; *Niebezpieczne objawy w szkolnictwie slaskiem*, ‘Gość niedzielny’ 1933, vol. 11, 1, 3–5.

91. APK, 27/200 (Ustawa w sprawie rozciągnięcia na obszar Województwa Śląskiego mocy obowiązującej niektórych przepisów ustawy z dnia 11 marca 1932 r. o ustroju szkolnictwa), 8.

92. J. Lusek, *Szkolnictwo niemieckie i polskie w Bytomiu w latach 1740–1945*, Opole 2010, 155–166.

93. Z. Osiński, *Janusz Jędrzejewicz – piłsudczyk i reformator edukacji (1885–1951)*, Lublin 2007, 197; K. Jędrzejczak, ‘Recepcja wychowania chrześcijańskiego w Polsce w rozumieniu Piusa XI’, in: E. Walewander (ed.), *Katolicka a liberalna myśl wychowawcza w Polsce w latach 1918–1939*, Lublin 2000, 451–455.

94. K. Sarzyński, ,Spór o wyznaniowy charakter szkół Górnego Śląska w dwudziestoleciu międzywojennym na podstawie „Gościa Niedzielnego” w latach 1923–1939‘, in: *Śląskie Studia Historyczno-Teologiczne* 47 (2014) 2, 346-360, 357; Glimos-Nadgórska, *Polskie*, 106; H. Czembor, *Ewangelicki Kościół Unijny na polskim Górnym Śląsku (1922–1939)*, Kraków 1993, 206–207.

95. Prażmowska, *Modern*, 124.

96. D. Wojtas, ‘Learning to Become Polish: Education, National Identity and Citizenship in Interwar Poland 1918–1939’, unpublished PhD thesis, Waltham 2003, 244.

97. Olszówka, ‘Szkolnictwo polskie’, 24; J. Kampczyk, ‘Adam Tyran (1882–1956)’, in: *Lubliniecki pamiętnik pedagogiczny* 2 (1997), 117–120, 118.

98. Janik, ‘Zespół’, 24.

99. H. Hein, *Der Piłsudski-Kult und seine Bedeutung für den polnischen Staat 1926–1939*, Marburg 2002, 14.

100. *Przegląd Polityczny*, 1932, vol. 16, 26–30.

101. A. M. Brzeziński, ‘Organizacja i formy międzynarodowej współpracy intelektualnej w ramach Ligi Narodów (1922–1939)’, in: *Przegląd Zachodni* 3 (2015), 7–23, 19.

102. Brzeziński, ‘Narodów’, 79.

103. H. Pohoska, *Dydaktyka historii*, Warsaw 1937 (second edition), 217.

104. Brzeziński, *Komisja*, 87.

105. Gelles, *Sprawy*, 191–197.

106. Markowiak, ‘Lublinieckie Gimnazjum’, 59.

107. Haubold-Stelle, *Mythos*, 242.

108. W. Liszkowski, ‘Szkoły i nauczyciele w moim zyciu‘, in: *Lubliniecki pamiętnik pedagogiczny* 1 (1994) 61–67, 65.

109. ‘Deklaracje polsko-niemieckie w sprawach mniejszościowych’, in: *Front Zachodni. Pismo poświęcone sprawom wzajemnego stosunku Polski i Niemiec* 5 (9.11.1937), 1; APK, 1363/413 (Znaczenie wpisów wobec wygasania Konwencji Genewskiej i sposoby ich przeprowadzenia, 1937), 8–9.

110. BABL, 649 (Kattowitzer Lehrplan für Geschichte am Gymnasium, 1.12.1936, Anlage 6: Geschichte), 1–7.

111. BABL, 647 (Die Feststellung des nicht zureichenden Unterrichtsniveaus an den Gymnasien Tarnowitz und Pless, 1937), 5–6.

112. Ibid., 4–5.

113. T. Falęcki, *Niemieckie szkolnictwo mniejszościowe na Górnym Śląsku w latach 1922–1939*, Katowice 1970, 151–152.

